# An Altered DNA Methylation Status in the Human Umbilical Cord Is Correlated with Maternal Exposure to Polychlorinated Biphenyls

**DOI:** 10.3390/ijerph16152786

**Published:** 2019-08-04

**Authors:** Akifumi Eguchi, Shino Nishizawa-Jotaki, Hiromi Tanabe, Bahityar Rahmutulla, Masahiro Watanabe, Hidenobu Miyaso, Emiko Todaka, Kenichi Sakurai, Atsushi Kaneda, Chisato Mori

**Affiliations:** 1 Center for Preventive Medical Sciences, Chiba University, Inage-ku Yayoi-cho 1-33, Chiba 263-8522, Japan; 2 Department of Bioenvironmental Medicine, Graduate School of Medicine, Chiba University, Chuo-ku Inohana 1-8-1, Chiba 263-8522, Japan; 3 Teijin Limited, Kasumigaseki Common Gate West Tower, 2-1, Kasumigaseki 3-chome, Chiyoda-ku, Tokyo 100-0013, Japan; 4 Department of Molecular Oncology, Graduate School of Medicine, Chiba University, Chuo-ku Inohana 1-8-1, Chiba 263-8522, Japan; 5 Department of Anatomy, Tokyo Medical University, Shinjuku-ku Shinjuku 6-1-1, Tokyo 160-8402, Japan

**Keywords:** polychlorinated biphenyl, birth cohort, DNA methylation

## Abstract

Maternal exposure to polychlorinated biphenyls (PCBs) results in abnormal fetal development, possibly because of epigenetic alterations. However, the association between PCB levels in cord serum with fetal DNA methylation status in cord tissue is unclear. This study aims to identify alterations in DNA methylation in cord tissue potentially associated with PCB levels in cord serum from a birth cohort in Chiba, Japan (male neonates = 32, female neonates = 43). Methylation array analysis identified five sites for female neonates (cg09878117, cg06154002, cg06289566, cg12838902, cg01083397) and one site for male neonates (cg13368805) that demonstrated a change in the methylation degree. This result was validated by pyrosequencing analysis, showing that cg06154002 (*tudor domain containing 9*: *TDRD9*) in cord tissue from female neonates is significantly correlated with total PCB levels in cord serum. These results indicate that exposure to PCBs may alter *TDRD9* methylation levels, although this hypothesis requires further validation using data obtained from female neonates. However, since the present cohort is small, further studies with larger cohorts are required to obtain more data on the effects of PCB exposure and to identify corresponding biomarkers.

## 1. Introduction

Undernutrition during gestation and early childhood is suggested to increase the risk of cardiovascular diseases in adulthood [1]; accordingly, the “Developmental origins of health and disease” (DOHaD) concept has been postulated [2]. The DOHaD paradigm encompasses nutritional deficiency, environmental stress, maternal stress, infection, and chemical exposure during gestation [3]. Epigenetic modifications during fetal development and childhood are potentially associated with lifestyle-related diseases in adults, indicating that the DOHaD paradigm may have an epigenetic mechanistic basis [4,5]. Exposure to persistent organic pollutants (POPs) was reported to be associated with global DNA hypomethylation in Greenland Inuit and healthy Korean populations [6,7,8]. Persistent organic pollutants including polychlorinated biphenyls (PCBs) may also affect DNA methylation, thus necessitating new risk assessment methods focusing on epigenetic alterations [9]. Epigenetic regulation is an important aspect of the DOHaD hypothesis [10], and several studies have reported epigenetic alterations during various developmental stages in response to maternal nutritional factors such as a high-fat diet [11], dietary folate, methionine, and vitamin B12 [12], and smoking habits [13]. Environmental chemical agents may also alter the degree of DNA methylation by interfering with histone modifications and the one-carbon and citric acid metabolism pathways [14,15,16].

PCBs are well-known endocrine disrupting chemicals and have been shown to persist in human tissues [17,18]. After the Yusho [19,20] and Yu-cheng incidents [21], neonates born to mothers exposed to PCBs and dioxin-like compounds had low birth weights [22]. In the Yu-cheng incident in Taiwan, the prevalence of physical and behavioral anomalies increased in PCB-exposed children, along with delayed physical and mental development [22]. Moreover, prenatal exposure to PCBs and polychlorinated dibenzofurans (PCDFs) was associated with alterations in DNA methylation levels in 11 genes in second-generation men after the Yu-cheng incident [23]. In the Yusho incident, developmental and reproductive impacts were observed in the exposed population [24,25,26].

PCBs have been detected in human umbilical cord (UC) tissue and cord blood in the general population [27,28], indicating that neonates may be exposed to these compounds via the maternal blood. Most of the previous studies focus on cord blood; however, as the structure of UC tissue is relatively uniform [29] and the cells comprising the cord tissue are of fetal origin, it may be appropriate for use in determining exposure outcomes in the context of toxico-epigenomic studies [30].

In this study, we analyze the relationship between PCB levels in cord serum and DNA methylation degrees in the cord tissue using methylation array analysis. We validated the methylation degrees of the samples using pyrosequencing.

## 2. Materials and Methods

### 2.1. Ethics Statement

This study was approved by the Biomedical Research Ethics Committee of the Graduate School of Medicine, Chiba University (ID: 989). Informed consent was obtained from the study participants prior to the beginning of this study.

### 2.2. Subjects

This study involved one of the three hospital-based birth cohorts belonging to the Chiba study of Mother and Child Health (C-MACH). Since cord tissue sampling methods were not standardized, samples from only one hospital were used. This cohort from Onodera Ladies Clinic (Chiba, Japan) was used for investigating PCB levels in cord serum and DNA methylation degrees in cord tissue, with the aim of identifying potential biomarkers of PCB exposure. Cord serum samples and paired cord tissue (n = 93) were obtained from mothers who participated in this study. Smokers were excluded from this study. Table 1 summarizes the characteristics (sex, PCBs, methylation array data, and birth weight) of neonates (male neonates = 32, female neonates = 43) whose cord and cord serum samples and complete data from the neonate questionnaire (neonate sex and birth weight) were available. Table 2 summarizes the characteristics (neonate sex, age, height, body weight at 10 gestational weeks) of 68 subjects out of 93 study participants. Around 32 weeks of gestational age, expecting mothers were requested to complete questionnaires and to provide UC at the time of delivery. The UC blood samples were centrifuged to obtain the serum and stored at −80 °C until chemical analysis. Whole UC tissue was collected simultaneously and samples were transferred from the clinic to the laboratory. After washing the umbilical cord tissues with saline in the laboratory to remove cord blood, they were stored at −80 °C until DNA methylation analysis. Details of the C-MACH are given in our previous report [31].

### 2.3. PCB Analysis

Cord serum PCB levels of the participants were analyzed and reported in our previous study [32]. The levels of 16 kinds of PCB congeners (tetra- to nona-: PCB 66, 74, 99, 105, 118, 126, 138, 153, 156, 170, 177, 180, 183, 187, 194, and 201) were analyzed by gas chromatography (GC) negative ion chemical ionization quadrupole mass spectrometry (NICI-MS) in accordance with our previous study [33]. Briefly, 0.3–0.4 g of serum samples were denatured and extracted twice with 500 μL of *n*-hexane. After surrogates were spiked, the extracts were combined and washed with ultrapure water and the organic fraction was passed through a 44% sulfuric acid silica gel column. Cleaned samples were concentrated to almost completely dry and 20 pg of PBB 154 dissolved in 200 μL of decane was added as a syringe spike for GC-MS analysis [33]. The recovery rates of surrogates were calculated to monitor the method performance and adjust for sample loss in the sample pretreatment process and matrix effect in GC-MS analysis, and ranged from 70.4%–144%. These recovery rates were not used in calculating the PCB levels. The detection limits of the methods for individual PCBs were 1.9–20 pg/g wet wt. For quality assurance and control of PCB analysis, our group participated in an inter-calibration exercise organized by the NIST using the Standard Reference Material 1957 [32].

### 2.4. DNA Methylation Analysis

Methylation degree in the genomic DNA of UC tissues was analyzed using our previously used method [34]. Briefly, genomic DNA extracted from UC tissues was subjected to bisulfite modification using a NucleoSpin Tissue Kit (MACHEREY-NAGEL GmbH & Co. KG, Düren, Germany) and EZ DNA Methylation Gold Kit (Zymo Research, Los Angeles, CA, USA). The DNA methylation profiles of the UC tissues were determined using the Infinium Methylation EPIC BeadChip (Illumina, San Diego, CA, USA). The degree of methylation at each CpG, quantified with average β values, was calculated using GenomeStudio (Illumina, San Diego, CA, USA). β values were considered for data analysis.

### 2.5. Pyrosequencing Analysis

Quantitative validation for Infinium probes was performed via pyrosequencing as previously reported (Matsusaka et al., 2011). Briefly, genomic DNA (500 ng) obtained from each tissue sample was subjected to bisulfite modification and was eluted in 40 μL of 10 mM Tris buffer. Primers were designed by PyroMark Assay Design Software 2.0 (Qiagen, Hilden, Germany) to amplify bisulfite-treated DNA. The biotinylated PCR product was bound to Streptavidin Sepharose High Performance beads (Amersham Biosciences, Uppsala, Sweden), washed, and denatured using 0.2 M NaOH solution. After addition of 0.3 μmol/L sequencing primer to the single stranded PCR product, pyrosequencing was carried out using a PyroMark Q96 ID system (Qiagen, Hilden, Germany) in accordance with the manufacturer’s instructions. Details of the primers and probes used to investigate the methylation inversion are summarized in Table 3. To justify the assumption that the methylation differences were biologically relevant, the probe was not subjected to pyrosequencing analysis when the upper to lower range of DNA methylation degree was less than 0.1.

### 2.6. Data Analysis

All data analysis was performed using a statistical software package, R 3.5.1 [35]. The characteristics of participants of both sexes were compared using the Mann–Whitney U test. Methylated gene features with low variance (standard deviation <0.1) were initially removed from the dataset before modeling, which resulted in a reduction of the number of methylated gene loci from 865,918 to 13,850. The resultant number of loci were used for further data analysis. Correlations between DNA methylation degrees and PCB concentration in cord serum were analyzed by Spearman correlation. *p*-values were adjusted by the false discovery rate (FDR) using the R package psych [36,37] for data from the Infinium Methylation EPIC BeadChip. In this study, a value of <0.1 as the FDR was used as the threshold. Furthermore, the association between *β* values and log10-transformed PCB levels was analyzed using multiple linear regression analysis for loci validated via pyrosequencing.

Because all PCB congeners detected herein were strongly correlated, only the total PCB levels were considered for statistical analysis [32].

## 3. Results

The characteristics of the study participants are summarized in Table 1 and Table 2. The sum of the 16 PCB congeners (Total PCBs) were detected in all samples. There were no significant differences in total PCB levels (Male: Female, 72 [interquartile range, IQR: 52–120] and 66 [IQR: 44–100] pg/g wet, respectively), body weight at birth, and maternal characteristics between the male and female neonates (Table 1 and Table 2).

**Figure 1:**
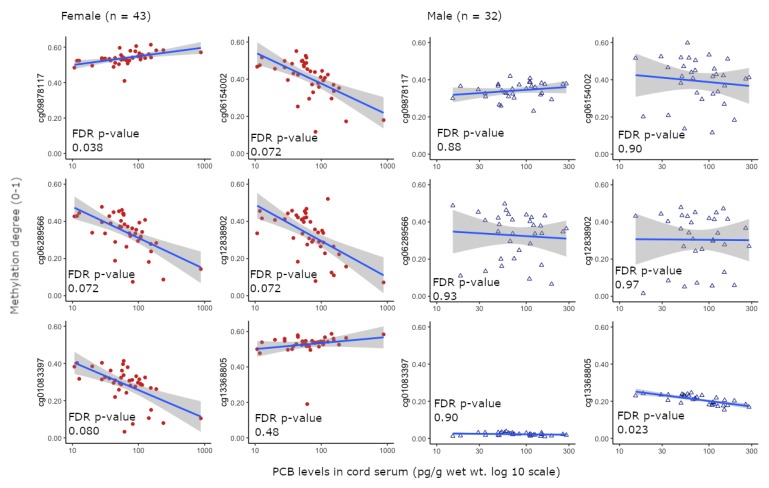
Relationship between maternal serum polychlorinated biphenyl (PCB) levels (pg/g wet weight) and DNA methylation degrees (0–1) six selected loci (cg09878117, cg06154002, cg06289566, cg12838902, cg01083397 and cg13368805) in cord tissue from male and female neonates revealed by correlation analysis (false discovery rate (FDR) < 0.1 in any one of gender) (gray 95% CI of single linear regression model). * Red circle ●: female newborns (n = 43: median 72 pg/g wet), blue triangle △: male newborns (n = 32: median 66 pg/g wet).

Correlation analysis indicated that total PCB levels in cord serum tended to be associated with the degree of methylation (FDR < 0.1) of genes in cord tissues at six sites: five in female neonates (cg09878117, cg06154002, cg06289566, cg12838902, cg01083397) and one in male neonates (cg13368805) (Figure 1, Table 3). Among these genes, cg09878117 was positively correlated, while cg06154002, cg06289566, cg12838902, and cg01083397 were negatively correlated with PCB levels in cord serum from female neonates (Figure 1, Table 4). In the six methylated locations, four gene names and groups (cg06154002 and cg06289566: *TDRD9* [TSS200]; cg12838902: *SLC29A4* [5’UTR; 1st exon]; cg01083397: *methyl-CpG binding protein 2*: *MECP2* [body; 5’UTR]; cg13368805: *ARAF* [TSS1500]) were annotated. Single nucleotide polymorphisms were present in cg06154002 and cg06289566 within 10 bp of the interrogated CpG site (rs113496080 and rs530246903), respectively (Table 4). The ranges of the upper to lower methylation degrees for five loci (cg09878117, cg06154002, cg06289566, cg12838902, cg01083397) in the cord samples from female neonates (around 20–50%) were higher than for male neonates (cg13368805: below 10%), and the ranges of methylation degree for three loci (cg06154002, cg06289566, cg12838902) and cg09878117 in both sexes were wide and low (range: around 50 and 20%, respectively) (Figure 1). Furthermore, the range of methylation degree of cg01083397 in male neonates was lower than that in female neonates.

Pyrosequencing analysis using the Infinium Methylation EPIC BeadChip to validate the results of correlation analysis indicated that only cg06154002 (tudor domain containing 9: TDRD9) was correlated with the total PCB level (Spearman correlation, *p*-value = 0.026, multiple linear regression [adjusted by age and BMI], *p*-value = 2.55e−05). The methylation levels determined via the EPIC array and pyrosequencing analysis in in cg06154002 and cg06289566 were correlated (Spearman correlation, *p* = 0.014 and 0.0081, respectively); however, other probes (cg09878117, cg12838902, cg01083397) were not significantly correlated (Spearman correlation, *p* = 0.064–0.71) (Figure S1). Because the upper to lower range of methylation levels (*β* values) in cord tissue samples obtained from male neonates were lower than the threshold of 0.1 (Figure 1), pyrosequencing analysis was not performed on these samples.

The present results show that DNA methylation is not significantly correlated with birth weight.

## 4. Discussion

Effects of exposure to chemicals, such as PCBs, dioxins, heavy metals, and bisphenol A, are associated with alterations in DNA methylation levels [8,38,39,40], possibly by mimicking the effects of endogenous hormones. However, limited information has been available regarding the association between chemical exposure and alterations in the DNA methylation levels in neonates. This study investigated the association between PCB levels in cord serum and DNA methylation degrees in UC to identify potential exposure markers and to compare the sex-specific difference of DNA methylation degrees.

Although background characteristics and PCB residue levels were not significantly different between male and female neonates, the number of methylated locations associated with PCB levels differed among female neonates (Table 1 and Table 2; Figure 1). Sex-dependent effects of PCB exposure have been reported in several studies [41,42,43,44,45]. An in vivo study reported sex-specific effects of perinatal dosing with a commercial mixture of PCBs (Aroclor 1254) on learning and sensory function in rats and reported that disruption of sex hormones is potentially associated with sex-specific effects [43]. Furthermore, Aroclor 1254 was a mixture of dioxin-like and non-dioxin-like PCBs [46], indicating that the association between PCB levels and methylation degree is potentially based on aryl hydrocarbon receptor (AhR)- and/or non-AhR-dependent effects. However, individual PCB congeners detected in more than 50% of the cord serum samples in the present study (CB74, 105, 118, 138, 146, 153, 170, 180, 183, 187, 194 and 201) were correlated with each other [32]. In the Yu-Cheng incident, the exposed male children attained lower scores on a cognitive development test (Colored Progressive Matrices and Standardized Progressive Matrices) than male controls, whereas no effects were observed in female children [47], suggesting possible sex-dependent differences in DNA methylation changes associated with PCB exposure.

In the present study, methylation array analysis of *TDRD9* revealed a positive correlation with residual levels of PCBs in female neonates (FDR: cg06154002 = 0.072, cg06289566 = 0.072), but not in male neonates (FDR: cg06154002 = 0.91, cg06289566 = 0.93). *TDRD9* is essential for silencing long interspersed element-1 (Line-1) retrotransposons, and that these transcripts are most abundantly elevated in meiotic spermatocytes in the mouse [48,49]. However, no discernible differences in meiotic progression or cellular defects were observed, consistent with the female fertility observed with these mutations [48]. Exposure of F0 generation rats to 2,3,7,8-tetrachlorodibenzo-*p*-dioxin (TCDD) causes the epigenetic transgenerational inheritance of adult-onset diseases, including prostate disease and polycystic ovarian disease in their offspring [39]. Furthermore, Su et al. reported that gestational levels of PCBs or PCDF toxic equivalency are significantly correlated with DNA methylation levels in AhR-related and carcinogenetic genes in second-generation men in the Yu-cheng incident [23]. However, exposure to non-AhR related compounds, such as methoxychlor and DDT, also alter the degree of DNA methylation related to male germline and ovarian function [50,51,52]. These results indicate that both dioxin-like compounds and non-AhR related endocrine disrupter might be related to reproductive dysfunction through alterations in the DNA methylation degree. The effect of alterations in the methylation degree of *TDRD9* on reproductive function in female neonates remains unclear.

Notably, sex-specificity was observed in the degree of DNA methylation at multiple loci (Figure 1), although a relationship with PCBs could not be clearly described due to lack of validation data (Figure S1). In particular, range of upper to lower methylation degree of cg01083397 (MECP2) in male neonates (below 10%) was lower than that in female neonates (around 40%) (Table 4 and Figure 1). *MECP2* has been linked to the X chromosome and causes Rett syndrome, a neurodevelopmental disorder [50]. Therefore, the relationship between PCB exposure and neurodevelopmental disorders in female newborns may be validated by increasing the number of study participants and analyzing them.

In our previous study, higher PCB levels in cord serum were related to low birth weight, synthesis of lipids, and fetal survival, growth, and health related biological pathways (amino-acid metabolism and ubiquinone and other terpenoid-quinone biosynthesis) in Japanese neonates [32]. However, no significant association between DNA methylation and birth weight was observed in the present study, probably owing to the small cohort size. Therefore, in future studies, a larger number of participants are required to yield more data on the PCB exposure effects and potentially identify biomarkers of PCBs exposure. Our present results suggest that in female neonates, exposure to PCBs in utero may alter the methylation degree of *TDRD9*, which is associated with reproductive function in men. Previous studies reported that both dioxin-like chemicals and non-AhR related chemicals may affect reproductive function through alterations in the DNA methylation degree [39,51,52,53]. However, the role of *TDRD9* in female neonates is still unclear; therefore, further studies are required to clarify the effect of alterations in the methylation degree of *TDRD9* in female neonates.

This study has several limitations. First, we excluded pregnant women with smoking habits from the analysis; however, the effect of second-hand smoke was not considered. We intend to analyze urinary cotinine levels in participants. Second, dietary profiles of participants were not considered herein, since these data have numerous variables. However, the main source of human exposure to PCB is diet, including fish, meat, egg, and dairy products [54]. We also previously reported that food consumption levels are associated with PCB levels in the body [55], indicating that the DNA methylation status is potentially associated with dietary habits. Third, we did not consider cell type specific DNA methylation. Lin et al. reported that DNA methylation profiles were cell-specific in cord tissue, and cellular heterogeneity should be adjusted for detail analysis [56]. Finally, the present cohort was relatively small; however, potential DNA methylation markers of PCB exposure were identified herein.

## 5. Conclusions

In conclusion, this study investigated the association between PCB levels in cord blood serum and DNA methylation levels in cord tissue. In this study, *TDRD9* methylation levels were analyzed via a methylation array and pyrosequencing and were correlated with PCB levels in cord blood serum in women. The role of *TDRD9* in female neonates is still unclear; therefore, future studies are required to clarify the effect of alterations in DNA methylation levels of *TDRD9* in female neonates.

## Figures and Tables

**Table 1:** Characteristics of newborn at Onodera Ladies Clinic (n = 75; data for gender, PCBs, methylation array data, and birth weight were available).

	Male	Female	*p*-Value
Gender of newborns (n)	32	43	
Total PCBs (pg/g wet wt) (median [IQR *])	72 [52, 120]	66 [44, 100]	0.247
Body weight at birth (g) (median [IQR])	2981 [2774: 3,164]	3052 [2898: 3195]	0.360

* IQR: interquartile range.

**Table 2:** Characteristics of mothers at Onodera Ladies Clinic (n = 68; data for gender of newborns, age, height and body weight at 10 gestational weeks were available).

	Male	Female	*p*-Value
Gender of newborns (n)	27	41	
Age (year) (median [IQR *])	33.0 [31.5, 36.0]	32.0 [31.0, 36.0]	0.431
Height (cm) (median [IQR])	159.0 [154.9, 164.7]	160.0 [156.0, 162.0]	0.945
Body weight at 10 gestational weeks (kg) (median [IQR])	53.0 [48.9, 56.5]	52.0 [48.0, 56.0]	0.716

* IQR: interquartile range.

**Table 3:** Primers for pyrosequencing.

Probe ID	MAP INFO	Strand	Primer Types	Primer Sequence	Anneal (°C)	Product (bp)
cg01083397	CHR X 153362990	Upper Strand	Forward *	GAGGGTAGAGAGGAGGGA	52	150
			Reverse	AACCAAAAAAAAAAACTATAAATAAAACC		
			Sequence	ACCCAAAAACCAAAATCAAAAA		
cg06154002	CHR 14 104394776	Upper Strand	Forward	GTTTTGATTGGAAGGTTT	54	125
cg06289566	CHR 14 104394782		Reverse *	TCCCCAACATCCTCAAAACCCA		
			Sequence	GGGAGGGGTTTTTAGGG		
cg09878117	CHR X 45649477	Lower Strand	Forward	GTTAGTTGGGTGGTTTTTTATGTTTTAG	54	138
			Reverse *	TCCACCCTATTTTTCCCAAAAC		
			Sequence	TGTTTTTGTTTATTTTTTAGAGTT		
cg12838902	CHR 7 5322586 (−200bp)	Lower Strand	Forward	GGGTTTTAGGGATAGGGG	52	106
			Reverse *	AAAACCTATCCAAACCTTTATCTAC		
			Sequence	GGGAGGGAGGGAGGGGG		
cg13368805	CHR X 47420179	Upper Strand	Forward *	ATTATAGTTTTTTAGAGTTGGTGAATA	54	117
			Reverse	CCAATACCCTAAACACCCTTTTAT		
			Sequence	CTTTAAAATAAATTAAAATCAAAAC		

Note: * indicates primers with 5’-biotin tag.

**Table 4:** Genes in cord tissue showing altered methylation degrees in relation to maternal PCB exposure according to correlation analysis.

FDR ^a^	Ilmn ID	UCSC RefGene Name ^b^	UCSC RefGene Group ^c^	SNP ID ^d^	SNP DISTANCE ^e^
Female: 0.038 (positive) Male: 0.88	cg09878117	NA	NA	NA	NA
Female: 0.072 (negative) Male: 0.90	cg06154002 ^‡^	*TDRD9*	TSS200	rs530246903; rs113496080	24;4
Female: 0.072 (negative) Male: 0.93	cg06289566	*TDRD9*	TSS200	rs530246903; rs113496080	30;10
Female: 0.072 (negative) Male: 0.97	cg12838902	*SLC29A4*	5’UTR;1st exon	NA	NA
Female: 0.080 (negative) Male: 0.90	cg01083397	*MECP2*	Body;5’UTR	NA	NA
Female: 0.48 Male: 0.023 (negative)	cg13368805	*ARAF*	TSS1500	NA	NA

^a^ False discovery rate of *p*-value (*p* < 0.1); ^b^ gene name (UCSC); ^c^ gene region feature category (UCSC); ^d^ IDs of SNPs within probe; ^e^ distance of SNPs within probe from query site; ^‡^ significantly correlated in pyrosequencing analysis (*p* < 0.05).

## Supplementary Materials

The following are available online at https://www.mdpi.com/1660-4601/16/15/2786/s1, Figure S1: Correlations between methylation array and pyrosequencing (Spearman’s correlation).
