# Supplementary material for: An Altered DNA Methylation Status in the Human Umbilical Cord Is Correlated with Maternal Exposure to Polychlorinated Biphenyls
Source: Int J Environ Res Public Health. 2019 Aug 4;16(15):2786. doi: 10.3390/ijerph16152786 (PMC6696183; doi:10.3390/ijerph16152786)
Supplement: Supplementary file 1 [file ijerph-16-02786-s001.pdf]

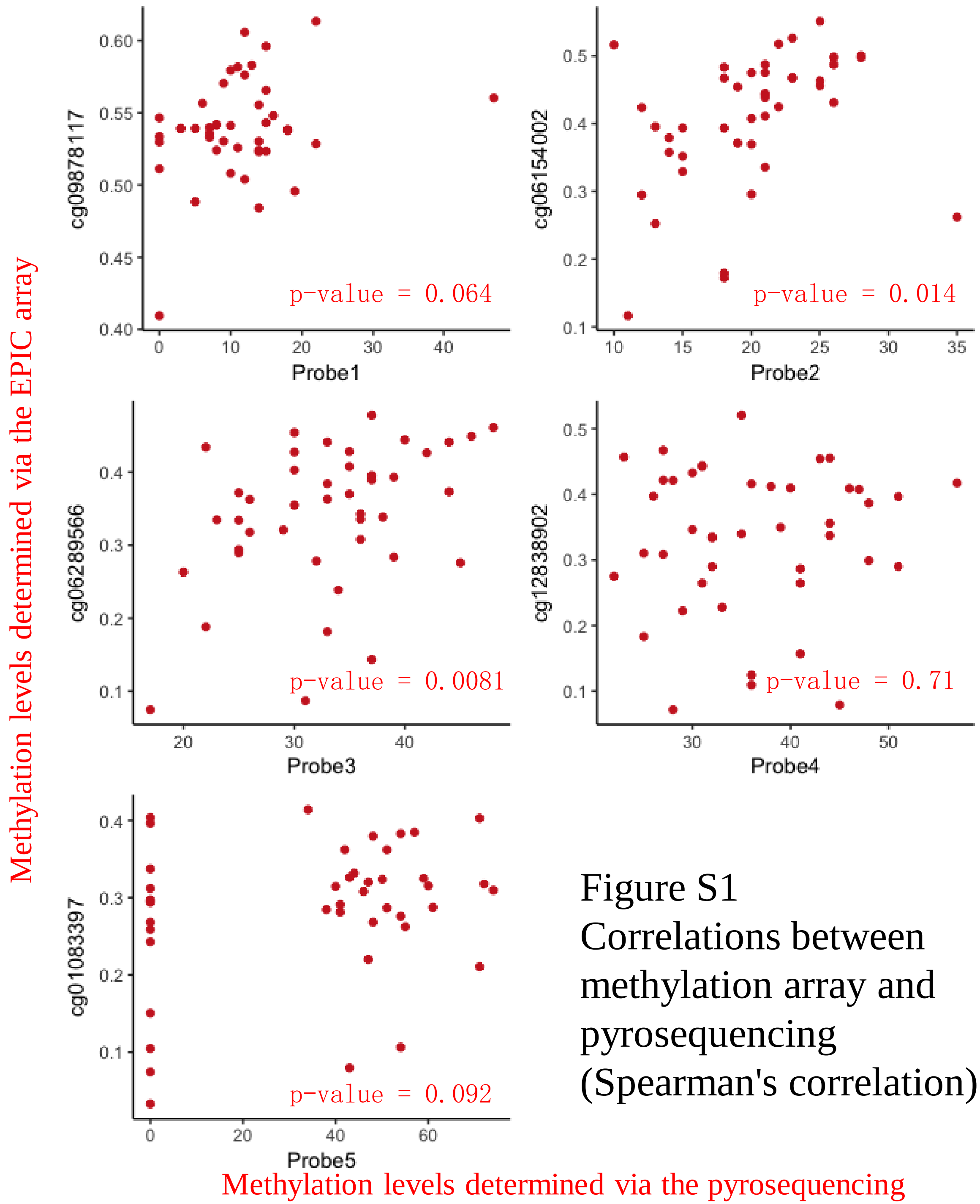

Figure S1
Correlations between methylation array and pyrosequencing (Spearman's correlation)
